# Unusual Presentation of Primary Pulmonary Sarcomatous Cancer With Brain Metastasis: A Case Report

**DOI:** 10.7759/cureus.51361

**Published:** 2023-12-30

**Authors:** Leena Alhusari, Ihab Tahboub, Moh'd Masoudi, Logan M Lawrence, Muhammad Jamil

**Affiliations:** 1 Internal Medicine Residency Program, Marshall University Joan C. Edwards School of Medicine, Huntington, USA; 2 Internal Medicine, Marshall University Joan C. Edwards School of Medicine, Huntington, USA; 3 Internal Medicine, Marshall University Joan C. Edwards School of Medicine, Edwards Comprehensive Cancer Center, Huntington, USA; 4 Pathology, Marshall University School of Medicine, Clinical Laboratories of the Mountain Health Network, Huntington, USA; 5 Hematology and Medical Oncology, Marshall University Joan C. Edwards School of Medicine, Edwards Comprehensive Cancer Center, Huntington, USA

**Keywords:** sarcomatoid variant, rare lung cancer, brain metastasis, pulmonary sarcomatous cancer, non-squamous cell lung cancer

## Abstract

Pulmonary sarcomatous carcinoma is a rare subtype of non-small cell lung cancer (NSCLC). This cancer has very low survival rates primarily due to its aggressive nature and propensity for early spread to abdominal organs and the skeletal system. Remarkably, brain metastasis is observed at later stages of the disease, likely attributing to the high fatality rate after the disease progresses to the brain tissue. In our case, a 79-year-old female with a 45-pack-year smoking history sought medical attention at a primary care clinic due to a 3-month history of recurrent right-sided chest pain. Notably, she denied cough, sputum production, palpitations, or syncope. CT chest revealed a 6.8 x 3.5 cm mass in the right upper lobe (RUL) of the lung, with evidence of obstruction and infiltration of the adjacent chest wall. A PET scan indicated increased uptake in the mass and the presence of smaller pulmonary nodules in both lungs, and multiple nodules in the upper left arm, abdomen, right inguinal region, left thigh, and cecum. Importantly, no intracranial lesions were detected. A subsequent colonoscopy yielded normal findings. Histopathologic examination of the lung mass and cell markers was consistent with a diagnosis of sarcomatous carcinoma of the lung. Only three days after the initial clinic visit, the patient presented with numbness and tingling in her lower extremities. Brain MRI revealed multiple bilateral brain metastases accompanied by significant vasogenic edema, prompting treatment with steroid therapy and brain radiation therapy. Subsequent chemotherapy/immunotherapy with Nab-paclitaxel /carboplatin/atezolizumab was initiated but led to significant treatment-related toxicities. Consequently, the treatment plan was adjusted to a single dose of single-agent immunotherapy using pembrolizumab. Unfortunately, the patient chose to discontinue treatment and eventually passed away after 13 days of palliative care. Compared to other lung cancer subtypes, brain metastasis in sarcomatous lung cancer is infrequent due to its lower prevalence among all lung cancer cases. Furthermore, sarcomatous lung cancer has a reduced propensity for developing brain metastasis when compared to other forms of non-small cell lung cancer (NSCLC). Regrettably, the prognosis for sarcomatous lung cancer with brain metastasis remains generally unfavorable, signaling an advanced stage of the disease with limited treatment options.

## Introduction

Sarcomatous carcinoma of the lung is a seldom encountered subtype of non-small cell lung cancer (NSCLC), constituting approximately 0.5% of all NSCLC cases [1,2]. Our case is consistent with the fact that this malignancy has an aggressive metastatic potential, resulting in a dismal prognosis, with a 5-year survival rate of merely 15-20% and a median survival duration of approximately 10 months [3,4]. The metastatic cells display a remarkable ability to disseminate through both lymphatic and blood vessels, giving rise to secondary tumors in various distant sites, including the gastrointestinal tract, kidneys, bones, and adrenal glands [5]. Notably, brain metastasis is unusual and considered a premature presentation in the sarcomatous variant of NSCLC. In this report, we present a compelling case of primary pulmonary sarcomatous carcinoma, characterized by its rapid metastasis to the brain.

## Case presentation

**Figure 1:**
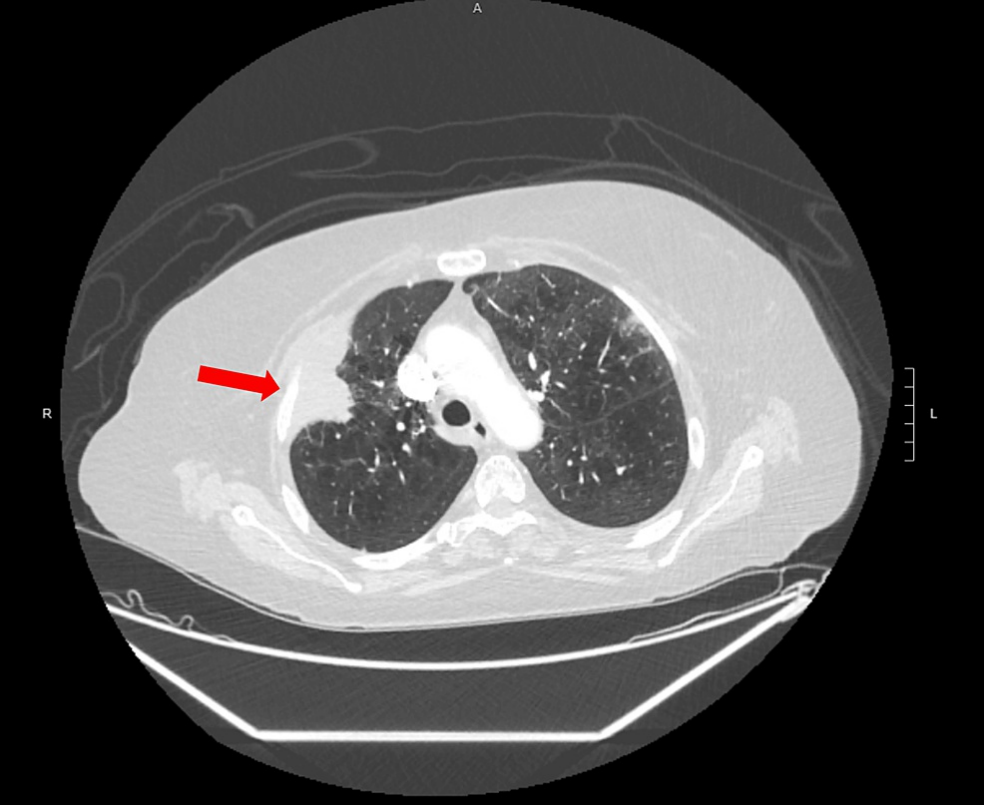
Chest CT scan showing a 6.8 x 3.5 cm mass in the right upper lobe of the lung

**Figure 2:**
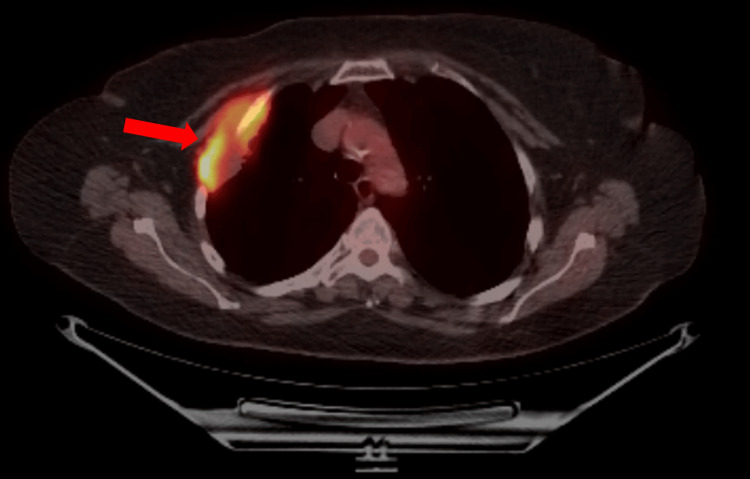
A PET scan showed increased uptake in the RUL mass

A 79-year-old Caucasian woman with a notable history of 45-pack-year smoking, chronic hypertension, and dyslipidemia, sought care at the clinic due to persistent right-sided chest pain for the last three months. Despite trying over-the-counter antacids, her symptoms had not improved significantly. She did not report any other respiratory issues, including cough, sputum production, palpitations, syncope, or constitutional symptoms. Radiological examinations, comprising a chest X-ray and subsequent chest CT scan, unveiled a peripheral mass in the right upper lobe (RUL) of the lung, measuring 6.8 x 3.5 cm, with evidence of infiltration into nearby chest wall structures, encompassing the pectoralis minor muscle and adjacent rib (Figure 1). A PET scan exhibited heightened uptake in the RUL mass and smaller bilateral pulmonary nodules (Figure 2).

**Figure 3:**
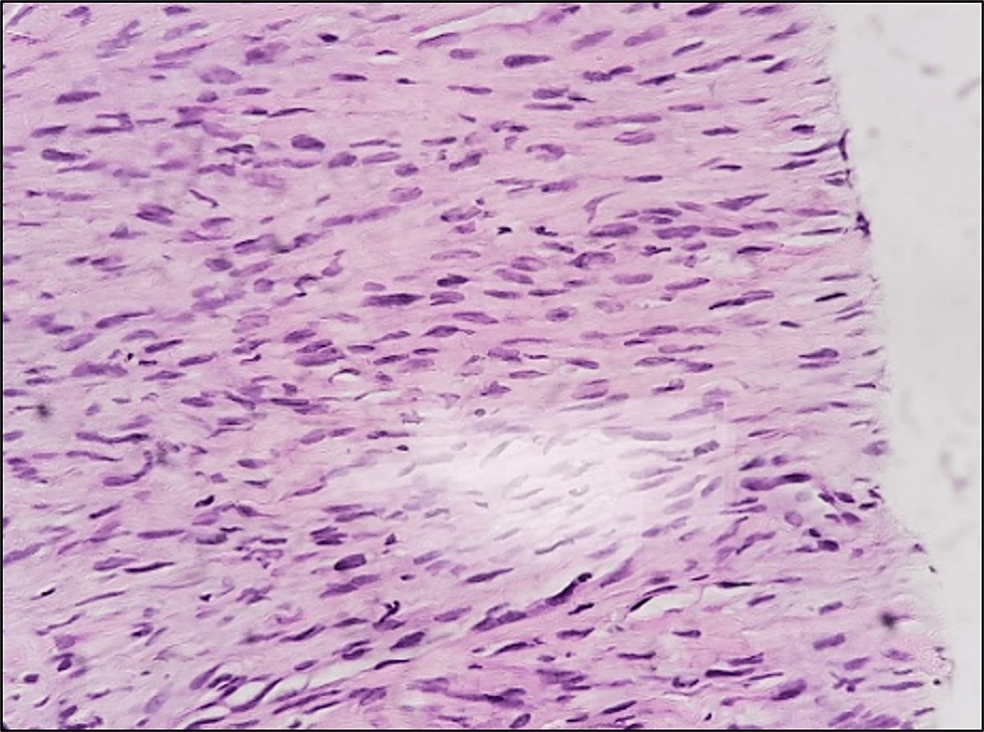
H&E stain showing solid sheets of moderately cellular pleomorphic spindle cell proliferation, focally necrotic. The spindle cells show an ill-defined cytoplasm and an ovoid nucleus with dense chromatin.

The PET scan additionally detected heightened uptake in multiple nodules situated in the left upper arm, abdomen, right inguinal region, left thigh, and cecum. Intriguingly, no intracranial lesions were observed. To explore the abdominal nodules further, a colonoscopy was conducted, yielding benign findings. A CT-guided biopsy of the lung mass unveiled a moderately cellular and pleomorphic spindle cell proliferation, featuring focal necrosis and ovoid nuclei with dense chromatin. These characteristics were consistent with a diagnosis of sarcomatous cancer (Figure 3).

**Figure 4:**
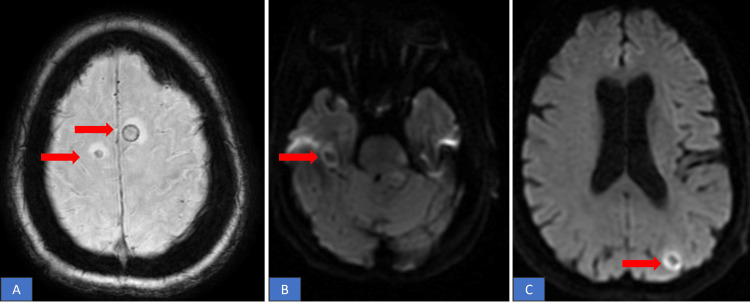
(a,b,c) MRI brain showing multiple enhancing foci noted bilaterally consistent with brain metastases.

**Figure 5:**
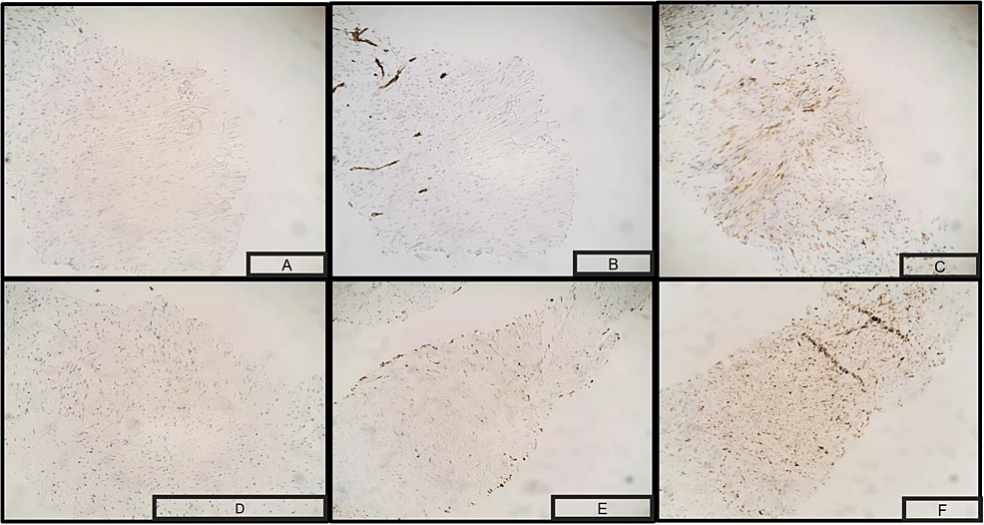
Tissue biopsy of the lung cancer testing positive for Immunostains suggestive of pulmonary sarcomatous cancer. A) positive S100 stain, B) positive p40 stain, C) cells positive for CD34 , D) cells positive for smooth muscle actin, E) positive Desmin stain, F) positive TTF-1.

Three days later, the patient revisited the clinic, reporting numbness and tingling in her lower extremities. Notably, she denied experiencing any other neurological symptoms, such as weakness, dizziness, imbalance, visual issues, or urinary or bladder incontinence. As part of the outpatient evaluation, an MRI of the brain revealed the presence of multiple bilateral brain metastases accompanied by vasogenic edema(Figure 4). The patient was admitted to the hospital for further assessment of these brain metastases and treated with steroids to alleviate the edema. Subsequently, the pathology was meticulously reviewed at Mayo Clinic, ultimately reaffirming the diagnosis of primary pulmonary sarcomatous carcinoma and was corroborated by the positive staining of tumor cells for pan-cytokeratin (AE1/AE3), CK7, and epithelial membrane antigen (Figure 5).

 

The patient underwent whole-brain radiation therapy followed by treatment with Nab-paclitaxel, carboplatin, and atezolizumab. Unfortunately, the patient encountered substantial treatment-related side effects, prompting a transition to single-agent immunotherapy involving pembrolizumab. After receiving a sole dose of pembrolizumab, the patient decided to discontinue aggressive treatment and opted for hospice care. Tragically, she passed away just 13 days later.

## Discussion

Sarcomatous carcinoma of the lung is distinguished by histological features reminiscent of sarcomas, marked by poorly differentiated cells with a spindle/giant-like appearance. The emergence of the sarcomatoid component in these tumors is believed to stem from cellular metaplasia secondary to the activation of an epithelial-mesenchymal transition program in a process called conversion paradigm [6]. The predominant carcinomatous component is typically squamous cell carcinoma (69%), followed by adenocarcinoma (20%), with large cell carcinoma being less frequent (11%) [7]. Primary pulmonary sarcomatous carcinoma (PSC) encompasses five major histological variants: pleomorphic, spindle cell, giant cell, carcinosarcoma, and pulmonary blastoma [8].

PSC is an exceedingly rare entity, representing less than 0.5% of all lung cancers [2]. Gender and heavy smoking are considered risk factors for the development of PSC with a male-to-female ratio of approximately 7:1 and occurring at a median age of 65 years [9]. The existing literature primarily relies on a limited number of case series and retrospective studies [10] hence, making it challenging to establish definitive clinical characteristics, diagnosis, treatment strategies, and survival outcomes [11].

PSC can manifest on imaging as a central mass with an endobronchial polypoid appearance or as a large peripheral mass with well-defined margins, often featuring necrotic, hemorrhagic areas, and occasionally cavitation [12]. Information regarding fluorodeoxyglucose (FDG) uptake in PET-CT imaging for these tumors is limited, with reports indicating significantly higher uptake compared to other lung cancer types or mentioning a maximum standardized uptake value (SUV) of 4.1 [13]. Tissue sampling of central masses are obtained endoscopically compared to transthoracic needling for peripheral lesions [14]. Obtaining a preoperative tissue diagnosis poses challenges due to the tumor's heterogeneity, as biopsies frequently represent only a single facet of the tumor [14]. Immunohistochemistry plays a pivotal role in PSC diagnosis. The histologic features include the presence of spindle or giant cells, along with at least 10% of the tumor mass exhibiting adenocarcinoma or squamous cell features [14]. PSC is associated with specific gene mutations (e.g., KRAS, P53, C-MET) which contribute to tumor aggression, frequent metastasis, and resistance to chemotherapy and tyrosine kinase inhibitors [8,12]. PD-L1 overexpression is often associated with the transition from adenocarcinoma histology to PSC [12].

The standard therapeutic approach for PSC remains undetermined due to its low incidence [15]. Sarcomas, including pulmonary sarcoma, often display resistance to chemotherapy with toxic chemotherapy being less effective compared to other sub-types of NSCLC. Hence, complete surgical resection is considered the most favorable treatment for early-stage PSC patients [16-18]. Alongside surgical intervention, radiation therapy is well-established and effective when combined with surgery [14]. While surgical resection can lead to long-term survival, considering adjuvant therapy is prudent due to the tumor's aggressive nature [19]. Chemotherapeutic agents such as doxorubicin, adriamycin, ifosfamide, and dacarbazine have proven effective. In cases of distant metastases, combination chemotherapy is recommended. Brain metastasis in sarcomatous lung cancer is relatively rare when compared to other types of non-small cell lung cancer (NSCLC) and other lung cancer subtypes. Recently introduced targeted therapies, such as those targeting epidermal growth factor receptor (EGFR) mutations and programmed cell death ligand-1 (PD-L1), have shown promise in individual cases [12].

Brain metastasis is found in roughly 3.3% of patients with sarcomas originating from skeletal or soft tissue, and the percentage is even lower when dealing with primary pulmonary sarcoma. Managing sarcomatous lung cancer with brain metastasis necessitates a multidisciplinary approach, involving surgery, radiation therapy, chemotherapy, targeted therapy, and immunotherapy, contingent upon the specific tumor characteristics and the patient's overall health [12]. Unfortunately, the prognosis for sarcomatous lung cancer with brain metastasis remains generally unfavorable, signifying an advanced stage of the disease with limited treatment options.

## Conclusions

Primary pulmonary sarcomatous carcinoma (PSC) stands as an infrequent subtype of NSCLC, characterized by a grim prognosis. Brain metastasis in sarcomatous lung cancer signifies an advanced disease stage. Diagnosing PSC can be intricate, necessitating extensive workup and immunohistochemistry for definitive confirmation. Encouragingly, targeted therapies and immunotherapy display the potential for enhancing PSC treatment outcomes, particularly in cases with specific mutations and PD-L1 expression. Further research and larger-scale studies are imperative to gain deeper insights into the molecular characteristics and optimize treatment strategies for this aggressive variant of lung cancer.

## References

[1] Franks TJ, Galvin JR (2010). Sarcomatoid carcinoma of the lung: histologic criteria and common lesions in the differential diagnosis. Arch Pathol Lab Med.

[2] Steuer CE, Behera M, Liu Y (2017). Pulmonary sarcomatoid carcinoma: An analysis of the National Cancer Data Base. Clin Lung Cancer.

[3] Maneenil K, Xue Z, Liu M (2018). Sarcomatoid carcinoma of the lung: The Mayo Clinic experience in 127 patients. Clin Lung Cancer.

[4] Goto T, Maeshima A, Tajima A, Kato R (2010). A resected case of pulmonary carcinosarcoma. Ann Thorac Cardiovasc Surg.

[5] Velcheti V, Rimm DL, Schalper KA (2013). Sarcomatoid lung carcinomas show high levels of programmed death ligand-1 (PD-L1). J Thorac Oncol.

[6] Pelosi G, Sonzogni A, De Pas T (2010). Review article: pulmonary sarcomatoid carcinomas: a practical overview. Int J Surg Pathol.

[7] Huwer H, Kalweit G, Straub U, Feindt P, Volkmer I, Gams E (1996). Pulmonary carcinosarcoma: Diagnostic problems and determinants of the prognosis. Eur J Cardiothorac Surg.

[8] Oliveira MF, Watanabe SC, Andrade MP, Rotta JM, Pinto FC (2013). Sarcomatoid carcinoma of the lung with brain metastases. J Bras Pneumol.

[9] Gleason T, Haas M, Le BH (2017). Imaging, histopathologic, and treatment nuances of pulmonary carcinosarcoma. Case Rep Radiol.

[10] Zhang MY, Tang LS, Qin ZJ, Hao YT, Cheng K, Zheng A (2022). Clinical features and prognostic factors of pulmonary carcinosarcoma: A nomogram development and validation based on surveillance epidemiology and end results database. Front Med (Lausanne).

[11] Litzky LA (2008). Pulmonary sarcomatous tumors. Arch Pathol Lab Med.

[12] Baldovini C, Rossi G, Ciarrocchi A (2019). Approaches to tumor classification in pulmonary sarcomatoid carcinoma. Lung Cancer (Auckl).

[13] Chuang TL, Lai CL, Chang SM, Wang YF (2012). Pulmonary carcinosarcoma: 18F-FDG PET/CT imaging. Clin Nucl Med.

[14] Panagiotopoulos N, Patrini D, Adams B (2016). Key features in the management of pulmonary carcinosarcoma. Case Rep Pulmonol.

[15] Karim NA, Schuster J, Eldessouki I (2018). Pulmonary sarcomatoid carcinoma: University of Cincinnati experience. Oncotarget.

[16] Braham E, Ben Rejeb H, Aouadi S, Kilani T, El Mezni F (2014). Pulmonary carcinosarcoma with heterologous component: Report of two cases with literature review. Ann Transl Med.

[17] Luh SP, Wu MH (20061). Pulmonary carcinosarcoma: Report of a case with review of the literature. Int Surg.

[18] Sayan M, Kankoc A, Ozkan D, Celik A, Kurul IC, Tastepe AI (2021). Prognostic analysis of primary pulmonary malignant mesenchymal tumors treated surgically. J Chest Surg.

[19] Venissac N, Pop D, Lassalle S, Berthier F, Hofman P, Mouroux J (2007). Sarcomatoid lung cancer (spindle/giant cells): An aggressive disease?. J Thorac Cardiovasc Surg.

